# Cardiomyocyte Proliferative Capacity Is Restricted in Mice With *Lmna* Mutation

**DOI:** 10.3389/fcvm.2021.639148

**Published:** 2021-06-23

**Authors:** Kenji Onoue, Hiroko Wakimoto, Jiangming Jiang, Michael Parfenov, Steven DePalma, David Conner, Joshua Gorham, David McKean, Jonathan G. Seidman, Christine E. Seidman, Yoshihiko Saito

**Affiliations:** ^1^Department of Cardiovascular Medicine, Nara Medical University, Kashihara, Japan; ^2^Department of Genetics, Harvard Medical School, Boston, MA, United States; ^3^Division of Cardiovascular Medicine, Howard Hughes Medical Institute, Brigham and Women's Hospital, Boston, MA, United States

**Keywords:** dilated cardiomyopathy, lamin A/C, cell cycle, p21, repressed proliferating capacity

## Abstract

*LMNA* is one of the leading causative genes of genetically inherited dilated cardiomyopathy (DCM). Unlike most DCM-causative genes, which encode sarcomeric or sarcomere-related proteins, *LMNA* encodes nuclear envelope proteins, lamin A and C, and does not directly associate with contractile function. However, a mutation in this gene could lead to the development of DCM. The molecular mechanism of how *LMNA* mutation contributes to DCM development remains largely unclear and yet to be elucidated. The objective of this study was to clarify the mechanism of developing DCM caused by *LMNA* mutation.

**Methods and Results:** We assessed cardiomyocyte phenotypes and characteristics focusing on cell cycle activity in mice with *Lmna* mutation. Both cell number and cell size were reduced, cardiomyocytes were immature, and cell cycle activity was retarded in *Lmna* mutant mice at both 5 weeks and 2 years of age. RNA-sequencing and pathway analysis revealed “proliferation of cells” had the most substantial impact on *Lmna* mutant mice. *Cdkn1a*, which encodes the cell cycle regulating protein p21, was strongly upregulated in *Lmna* mutants, and upregulation of p21 was confirmed by Western blot and immunostaining. DNA damage, which is known to upregulate *Cdkn1a*, was more abundantly detected in *Lmna* mutant mice. To assess the proliferative capacity of cardiomyocytes, the apex of the neonate mouse heart was resected, and recovery from the insult was observed. A restricted cardiomyocyte proliferating capacity after resecting the apex of the heart was observed in *Lmna* mutant mice.

**Conclusions:** Our results strongly suggest that loss of lamin function contributes to impaired cell proliferation through cell cycle defects. The inadequate inborn or responsive cell proliferation capacity plays an essential role in developing DCM with *LMNA* mutation.

## Introduction

Dilated cardiomyopathy (DCM), a significant cause of heart failure, is genetically inherited in 30 to 50% of cases (1, 2). *LMNA* is one of the leading causative genes of genetically inherited DCM as well as *TTN* or *MYH7* (2–4). Most DCM-causative genes (e.g., *TTN, MYH7*, and *TNNT2*) encode sarcomeric proteins or sarcomere-related proteins and are directly involved in the generation or transmission of the contractile force of the cardiomyocyte. Unlike these sarcomere-related genes, *LMNA* encodes nuclear envelope proteins, lamin A and C, and does not directly associate with contractile function. However, a mutation in this gene could lead to the development of DCM (4, 5), which in many cases is also accompanied by defects in the conduction system (2, 6) and poor prognosis (7). Moreover, mutations in *LMNA* are also known to cause a range of diseases, including myopathies and neuropathies such as limb-girdle muscular dystrophy (8), Emery-Dreifuss muscular dystrophy (9), Charcot-Marie-Tooth neuropathy (10), and Hutchinson-Gilford progeria (11).

Lamin proteins are structural proteins of the inner nuclear membrane, and an *LMNA* mutation is reported as the cause of morphological changes in nuclei such as flattening and bleb formation (12–15). Nikolova et al. reported the cardiac phenotypes resulting from this mutation using *Lmna* homozygous knockout (*Lmna*^−/−^) mice, which presented left ventricular (LV) dilation, reduced systolic function, and died around 6 weeks. *Lmna*^−/−^ myocytes showed altered nuclear shapes, decreased size, impaired contractility, and diminished Ca^2+^ binding affinity to myofilament (13). Morales Rodriguez et al. reported the altered calcium cycling could be related to LV dysfunction (16). Wolf et al. reported the impaired contractility of myocytes accompanied by atrioventricular conduction defects in *Lmna* heterozygous knockout (*Lmna*^+/−^) mice, mimicking human patients' phenotypes (17). Macquart et al. proved that altered distribution of a major gap junction protein Cx43 contributes to conduction defects using *Lmna* mutant mice (18). Thus, the molecular mechanism of how *LMNA* mutation contributes to DCM development is gradually elucidated, however, there remain unclear mechanisms to be elucidated.

Recently, cell cycle activity is reported to be related to the phenotype of the mammalian heart (19, 20). The development of the mammalian heart is characterized by cardiomyocyte proliferation and hypertrophy (21). In most mammalian hearts, cardiomyocytes proliferate before birth, followed by exiting the cell cycle by the postnatal change of nutrients, increased hemodynamic stress, and increased oxygen concentration (22, 23). Before exiting the cell cycle, murine neonatal cardiomyocytes undergo karyokinesis without cytokinesis from around 4 days after birth (21, 24), making the number of nuclei double per cell. By 12 days after birth, approximately 90% of the cardiomyocytes are binuclear, which is the physiological termination of the cell cycle. The heart grows mainly with hypertrophy of the cardiomyocyte after this period. When this physiological cell cycle activity is impaired by some reasons such as preterm birth, cardiac morphological change is observed (25, 26), and sometimes cardiomyopathy develops (27). On the contrary, under the condition in which the cell cycle is activated, the heart proliferates too much and sometimes develops hypertrophic phenotype as previously reported in the mouse with *MYBPC3* mutation (28) or hypoxic condition (19). Thus, the cell cycle activity is essential in developing the heart and could influence cardiac morphology. This cell cycle activity is reported to be retarded in lamin A/C-depleted cells (29) and skeletal muscle in *Lmna*^−/−^ mice (30). However, the cell cycle activity in the heart under the physiological condition, stress, and its relation to the development of DCM in *Lmna* mutant mice has not been elucidated to date.

We, therefore, studied the cell cycle activity in the mouse model with *Lmna* mutation and pursued the mechanism of developing DCM caused by *LMNA* mutation.

## Materials and Methods

All experimental protocols for the animal models were approved by the Animal Care and Use Committee of Harvard Medical School (#2530) and Nara Medical University (#11251, #11355).

### Mice

*Lmna* mutant mice were generated by the deletion of exon 8 to 11 of *Lmna* as described before (12). In brief, the targeting vector, removing exon 8 to 11 of *Lmna*, was electroporated into W9.5 embryonic stem cells. Two clones were injected into C57BL/6 blastocysts, and chimeras were bred to produce germline offspring. This line was backcrossed to 129S6/SvEvTac strain, obtained from Taconic Biosciences Inc. (Rensselaer, NY, USA), for more than 10 generations. Mouse genotypes were determined by PCR amplification of tail genomic DNA.

### Echocardiographic Studies

Mice were anesthetized with 1–1.5% inhalational isoflurane. Each limb was placed on the ECG leads on a Vevo Mouse Handling Table (FujiFilm VisualSonics Inc., Toronto, ON, Canada), maintaining the body temperature at 37°C during the study. Transthoracic echocardiography was performed using a Vevo 770 High-Resolution *In vivo* Micro-Imaging System and RMV 707B scan-head (FujiFilm VisualSonics Inc.) with heart rate at 450–550 beats per minute. The images were acquired as 2-dimensional mode (left parasternal long and short axes) and M-mode (left parasternal short axis). Measurements averaged from 3 consecutive heartbeats of M-mode tracings were used for LV wall thickness, LV end-diastolic, and end-systolic diameters. All echocardiographic measurements were done blinded to mouse genotype.

### EdU Cell Proliferation Assay

The thymidine analog 5-ethynyl-2′-deoxyuridine (EdU) cell proliferation assay was performed using Click-iT® EdU Alexa Fluor® 594 Imaging Kit (Life Technologies, Carlsbad, CA, USA). EdU is incorporated into DNA during DNA synthesis, the same as 5-bromo-2′-deoxyuridine (BrdU). EdU (5 μg/g body weight) was injected intraperitoneally for 5 consecutive days starting from the day of birth. Mice were sacrificed at 3 weeks of age and processed for EdU detection following the company's protocol.

### Apical Resection

LV apical resection procedure was performed as described before (31). In brief, mice at the age of 2 days after birth were anesthetized on ice to induce sedation. The thoracic cavity was opened at the fourth intercostal space, and the exposed LV apex was then resected. Following surgery, mice were warmed up to 37°C and were monitored for viability. Sham-operated mice underwent identical procedures without LV apical resection. Five μg/g body weight of EdU was administered subcutaneously after the surgery for 10 days every 2 days. Mice were euthanized at 3 weeks of age under 4–5% continuous inhalational isoflurane by exsanguination to assess cardiomyocyte proliferation.

### Histological Examination

To quantitate the number of nuclei per myocyte, isolated myocytes were labeled with 4′,6-Diamidine-2′-phenylindole dihydrochloride (DAPI, Sigma-Aldrich, St. Louis, MO, USA) and counterstained with anti-troponin I antibody (Ab) (1:200 dilution, Abcam, Cambridge, UK). To quantify cell cycle activity, isolated myocytes were labeled with anti-phospho-histone H_3_ Ab (Ser10, 1:100 dilution, Millipore, Billerica, MA, USA). Four percentage PFA-fixed paraffin-embedded hearts were cross-sectioned in 4 μm thickness and stained with hematoxylin and eosin and Masson's trichrome staining. The tissue sections were stained with Alexa Fluor® 488 or 594 conjugate of wheat germ agglutinin (WGA, Life technologies) for cell number counting and cell size measurement. To quantify the number of myocytes in tissue sections, cell counts were obtained from 10 different layers of the heart. We utilized Image J (NIH software) for counting cell numbers and measurement. Immunostaining for p21 was performed with anti-p21 Ab (ab2961, 1:40 dilution, Abcam). We applied anti-phospho-histone H_2_A.X Ab (pH_2_AX; Ser139, 1:480 dilution, Cell Signaling Technology, Danvers, MA, USA) to detect double-strand DNA break. EdU detection was performed in either 4% PFA-fixed frozen tissues embedded in optimal cutting temperature (OCT) compound (Sakura Finetek, Tokyo, Japan) or 10% Formalin-fixed paraffin-embedded tissue. Counterstaining was done with DAPI. Immunostaining positive cells or the number of nuclei were counted independently by two authors blinded to specimens' background.

### Protein Analysis

Western blot analyses were performed as in the conventional method. Briefly, total protein was extracted from frozen tissue, separated by SDS-PAGE, and hybridized with primary anti-p53 Ab (sc-100, 1:200 dilution, Santa Cruz Biotechnology, Dallas, TX, USA), anti-p21 Ab (sc6246, 1:200 dilution, Santa Cruz Biotechnology), or anti-β actin Ab (ab8227, 1:1000 dilution, Abcam). Hybridized signals were quantified by Image J and normalized to β actin.

### RNA-Sequence and Data Analysis

Total RNA was extracted from flash-frozen heart specimens with Trizol (Life Technologies), followed by mRNA purification by poly-A selection and cDNA synthesis using standard protocols to construct RNA-seq libraries as reported previously (32). The index was added to cDNA to distinguish samples. 20 nmol of each library was analyzed using the next-generation sequencing platform, Illumina HiSeq 2000 (Illumina, San Diego, CA, USA). Paired-end, 50 bp reads were aligned to mouse genome mm9 using TopHat ver1.4 (http://tophat.cbcb.umd.edu/). Gene expression profiles were generated as described before using a Bayesian *P*-value (33). The number of reads was normalized to total aligned reads on gene loci per 1 million reads. RNA-seq results were analyzed by IPA (http://www.ingenuity.com). Up- or down-regulated gene was defined as having more than or less than 1.5-fold transcriptional level compared to age-matched wild type (wt). Genes with expression values <1 read per 1 million reads (as evaluated in the wt sample) were excluded from the analysis.

### Statistics

Continuous data are expressed as mean ± SD. The significance of differences between two groups was determined using the Student's T-test, and that between more than 3 groups was determined with 1-way ANOVA. *Post-hoc* pairwise comparisons were performed with the Tukey-Kramer test. *P* < 0.05 were considered statistically significant. The *P-value* associated with functional analysis in IPA was calculated with the right-tailed Fisher's exact test.

## Results

### Small Heart Phenotype in *Lmna^−/−^* Mouse Is Caused by the Reduction of Both the Number and the Size of Cardiomyocytes

As reported previously (34), 2-year old *Lmna*^+/−^ mice were observed to have dilated LV in both systolic and diastolic phases and reduced LV contraction, whereas 5-week and 1-year-old *Lmna*^+/−^ mice did not present a DCM phenotype yet (Table 1). Overall, *Lmna*^−/−^ mice were smaller in body size than their age-matched wt and *Lmna*^+/−^ littermates. LV chamber size was enlarged in both systolic and diastolic phases after body surface area correction, and LV contractile function was reduced in *Lmna*^−/−^ mice compared to their controls. Masson's trichrome staining showed massive fibrosis in *Lmna*^−/−^ mice at 5 weeks of age (Supplementary Figure 1). Interestingly, those cardiac phenotypes such as enlarged LV chamber, reduced contractile function, and severe fibrosis were comparable to those of *Lmna*^+/−^ mice observed at 2 years of age (Table 1, Supplementary Figure 1), which suggested that the cardiac phenotypes developed more rapidly in *Lmna*^−/−^ mice than in *Lmna*^+/−^ mice, sharing similar pathology at different ages. Thus, the *Lmna*^−/−^ mice offer a model for studying DCM that rapidly develops within the lifespan of the mice. Identifying the molecular determinants of DCM and morphological characteristics in these mice allows for opportunities to understand and potentially pharmacologically treat the pathology in both *Lmna*^−/−^ and *Lmna*^+/−^ mice, with extensions to understanding how DCM develops in humans with *LMNA* mutations, which typically appear as autosomal dominant heterozygous mutations.

**Table 1:** Physical and echocardiographic characteristics of the mice used in this study.

**Age group**	**0 year**			**1 year**		**2 years**	
	**(5 weeks old)**			**(43–53 weeks old)**		**(86–100 weeks old)**	
**Genotype**	**wt**	***Lmna*^**+/−**^**	***Lmna*^**−/−**^**	**wt**	***Lmna*^**+/−**^**	**wt**	***Lmna*^**+/−**^**
	**(*n* = 6)**	**(*n* = 8)**	**(*n* = 5)**	**(*n* = 5)**	**(*n* = 9)**	**(*n* = 6)**	**(*n* = 10)**
BW (g)	18.1 ± 2.5	19.7 ± 2.7	8.5 ± 1.7^†^	34.5 ± 2.0	33.5 ± 3.3	36.5 ± 7.1	34.4 ± 7.8
HW/BW (mg/g)	5.8 ± 0.4	5.6 ± 0.6	6.5 ± 1.1	5.0 ± 0.7	4.6 ± 0.4	5.7 ± 1.0	8.9 ± 2.9^†^
IVS (mm)	0.64 ± 0.03	0.63 ± 0.05	0.45 ± 0.08^†^	0.76 ± 0.05	0.73 ± 0.05	0.82 ± 0.14	0.74 ± 0.08
LVDd (mm)	3.03 ± 0.28	3.25 ± 0.22	2.62 ± 0.27^*^	3.60 ± 0.32	3.54 ± 0.33	4.10 ± 0.26	4.81 ± 0.71
LVDd/BSA (mm/m^2^)	924 ± 99	934 ± 66	1,331 ± 163^†^	709 ± 60	713 ± 77	778 ± 115	977 ± 234
LVDs (mm)	1.82 ± 0.18	1.82 ± 0.36	1.81 ± 0.43	2.33 ± 0.38	2.09 ± 0.25	2.78 ± 0.46	3.99 ± 0.80^†^
LVDs/BSA (mm/m^2^)	555 ± 67	520 ± 81	914 ± 182^†^	458 ± 61	421 ± 62	524 ± 89	817 ± 251^†^
PW (mm)	0.62 ± 0.06	0.64 ± 0.06	0.48 ± 0.03^†^	0.88 ± 0.05	0.79 ± 0.10	1.03 ± 0.11	0.77 ± 0.11^†^
FS (%)	40.0 ± 3.8	44.1 ± 9.1	31.6 ± 9.0	35.2 ± 9.1	40.9 ± 7.0	32.3 ± 9.7	17.5 ± 7.0^†^

*BSA, body surface area; BW, body weight; FS, fractional shortening; HW, heart weight; IVS, interventricular septum; LVDd, diastolic left ventricular diameter; LVDs, systolic left ventricular diameter; PW, posterior wall; FS was calculated by [(LVDd-LVDs)/LVDd] × 100. LV mass was calculated by (IVS + PW + LVDd)^3^-LVDd^3^.*

* *p < 0.05 vs. wt,*

† *p < 0.01 vs. wt, Student's 2-tailed T-test*.

**Figure 1:**
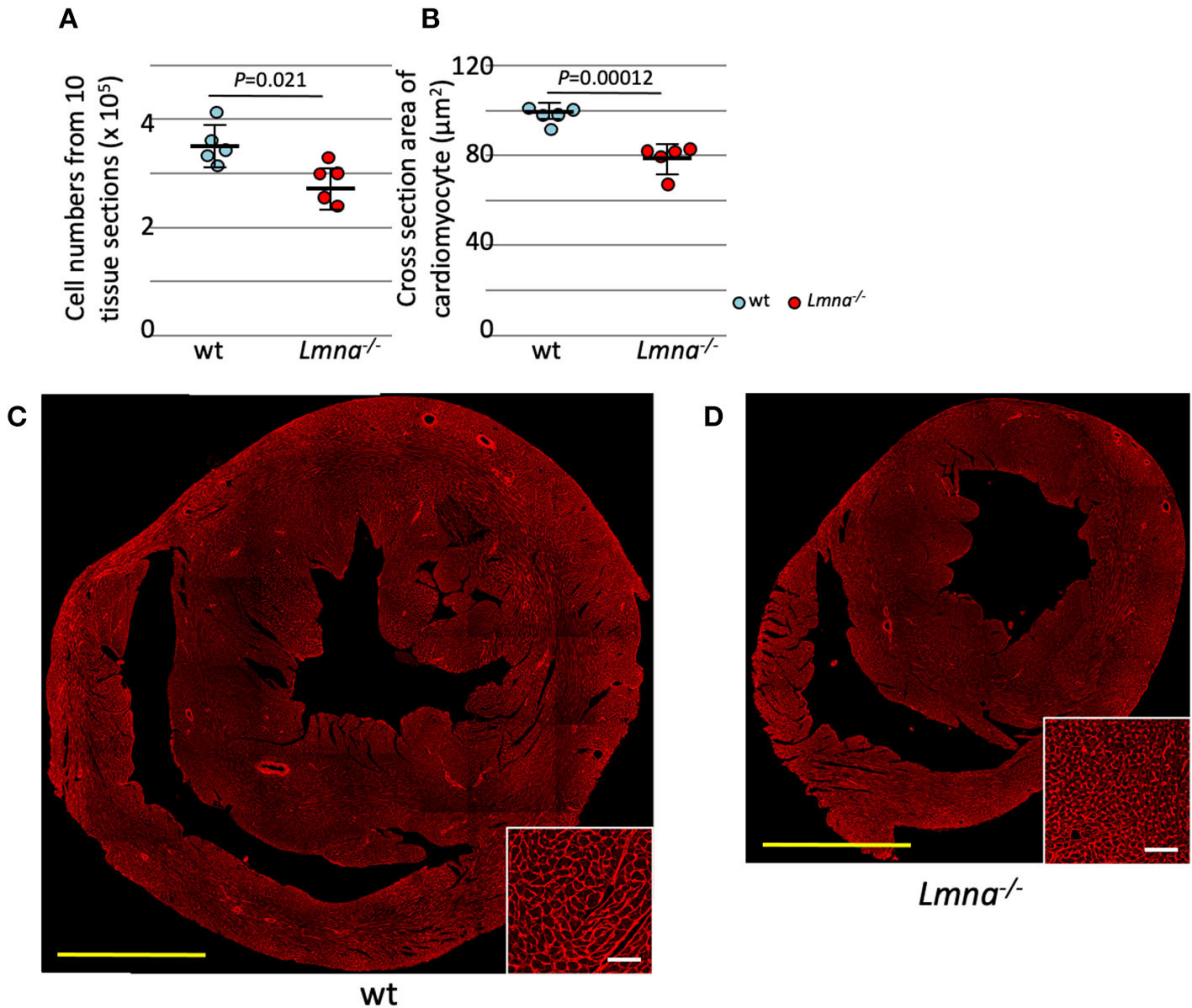
Cardiomyocyte cell number and size in wild type (wt) and *Lmna*^−/−^ mice. Both the cell number and size of cardiomyocytes were significantly reduced in *Lmna*^−/−^ mice (3 weeks old). **(A)** Total counts of cardiomyocytes in 10 tissue layers of the heart (*n* = 5 in each group). **(B)** Cross-section area of cardiomyocytes measured in a tissue section (*n* = 5 in each group). **(C,D)** Tissue sections stained with wheat germ agglutinin in wt **(C)** and *Lmna*^−/−^ **(D)** mice. The open circle represents wt, and the solid circle represents *Lmna*^−/−^ mice. The significance of differences between two groups was determined using the Student's 2 tailed *T*-test. Scale bar: 1 mm in lower magnification and 50 μm in higher magnification in inset.

The total number of myocytes was counted in 3-week-old wt and *Lmna*^−/−^ mice. Myocyte counts from tissue sections of 10 levels from apex to the base sequentially with the distance of 100 μm between each level (2.85 ± 0.37 × 10^5^ in *Lmna*^−/−^ and 3.54 ± 0.39 × 10^5^ in wt, *P* = 0.021; Figure 1A) showed a significant decrease in the number of *Lmna*^−/−^ myocytes. Moreover, myocyte cell size in tissue sections was significantly reduced in *Lmna*^−/−^ hearts (75.1 ± 6.5 μm^2^ in *Lmna*^−/−^ and 100.9 ± 5.2 μm^2^ in wt, *P* = 0.00012, Figures 1B–D insets). These results indicated a defect in both proliferation and growth of cardiomyocytes in *Lmna*^−/−^ mice.

### Retardation of Nucleation and Cell Cycle Activity in *Lmna^−/−^* Mice

**Figure 2:**
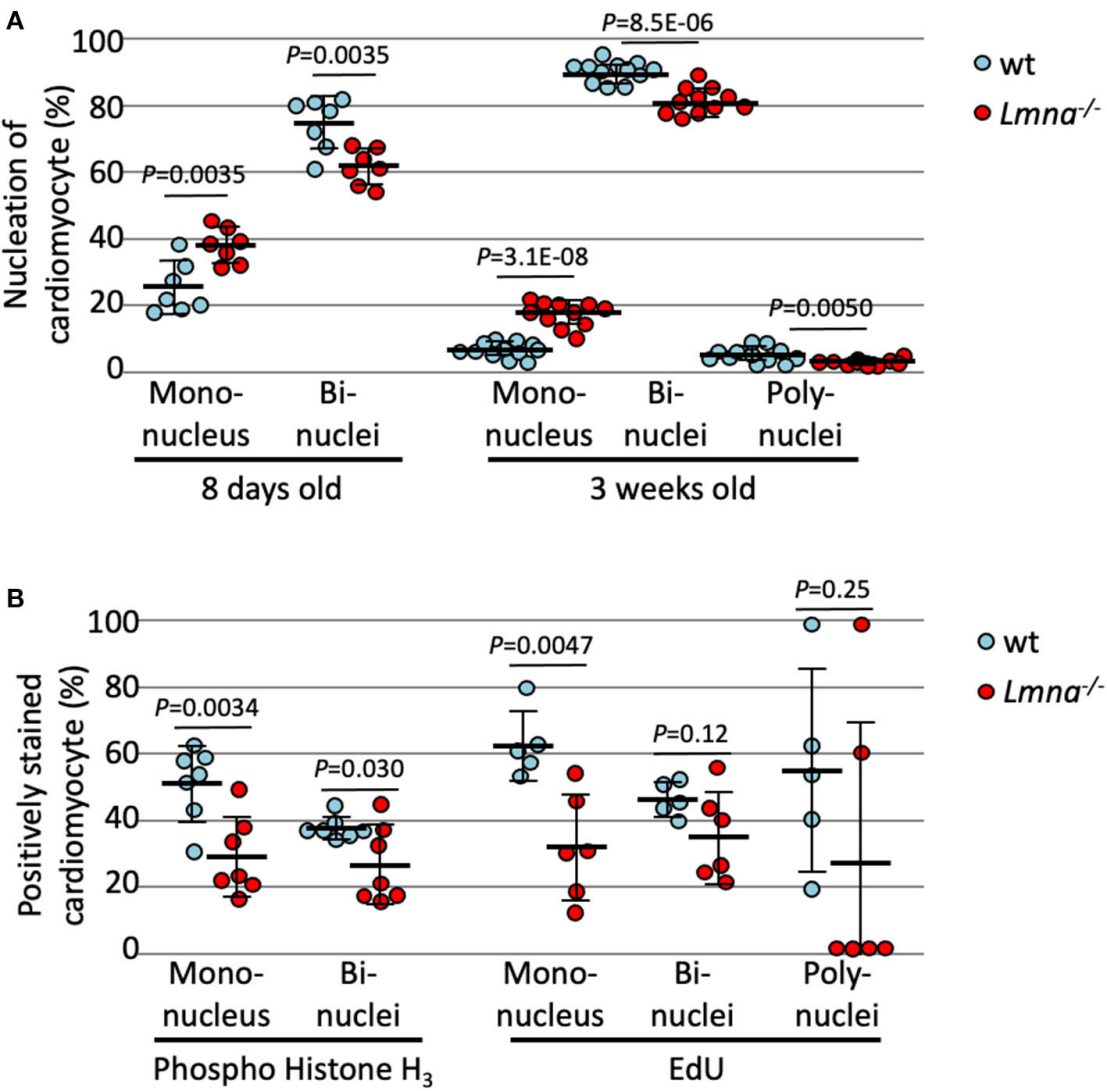
Nucleation and cell cycle markers of cardiomyocytes in wild type (wt) and *Lmna*^−/−^ mice. **(A)** Nucleation of cardiomyocytes in 8 days old (*n* = 7 in each group) and 3 weeks old (*n* = 12 in wt and 11 in *Lmna*^−/−^) showed more mononuclear and less bi- or poly-nuclear cardiomyocytes in *Lmna*^−/−^ mice. **(B)** Percentage of phospho-histone H_3_ positive cardiomyocytes in 8 days old (*n* = 7 in each group) and of EdU positive cells in 3 weeks old (*n* = 5 in wt and 6 in *Lmna*^−/−^) showed retarded cell cycle in *Lmna*^−/−^ mice. Dots represent individual mouse data. The significance of differences between two groups was determined using the Student's 2 tailed *T*-test.

As previously mentioned, murine cardiomyocytes transition to binuclear from mononuclear ~4 days after birth, and by 12 days, more than 90% of myocytes become binuclear. We hypothesized that binuclear or multinuclear cardiomyocytes are mature cells, while mononuclear cells are immature, and assessed the maturity of cardiomyocytes by counting the number of nuclei per cell. At 8 days after birth, *Lmna*^−/−^ had a significantly higher number of mononuclear myocytes compared to wt (37.9 ± 5.4% in *Lmna*^−/−^, 24.7 ± 8.0% in wt, *P* = 0.0035, *n* = 7 in each group, approximately 250 myocytes were counted in each mouse, Figure 2A), in consequence, binuclear cell counts were reduced in *Lmna*^−/−^ compared to wt. At 3 weeks after birth, mononuclear myocytes were observed 16.6 ± 3.9% in *Lmna*^−/−^ but only 5.6 ± 2.2% in wt (*P* = 3.1 × 10^−8^, *n* = 11, and 12, respectively, Figure 2A), while binuclear cells were 81.7 ± 4.1% in *Lmna*^−/−^ but 90.4 ± 3.0% in wt (*P* = 8.5 × 10^−6^, Figure 2A). Furthermore, 4.0 ± 2.2% of myocytes in wt presented more than 3 nuclei, whereas only 1.7 ± 0.9% of myocytes were multinuclear in *Lmna*^−/−^ (*P* = 0.0050, Figure 2A). These results suggest that the lack of *LMNA* retards the cardiomyocyte maturity.

To exclude the possibility that increased mononuclear cells in *Lmna*^−/−^ myocytes are the results of the completion of cell division rather than immaturity (28), we evaluated the cell cycle status of each cardiomyocyte with immunocytochemistry of phospho-histone H_3_ (pH_3_), which is a mitosis phase marker, and EdU cell proliferation assay, which is a synthesis phase marker. As shown in Figure 2B, reduced pH_3_ positive cardiomyocytes at 8 days after birth were observed in *Lmna*^−/−^ compared to wt in both mononuclear and binuclear myocyte populations (28.3 ± 12.1% vs. 51.0 ± 11.3%, *Lmna*^−/−^ and wt respectively, in mononuclear myocyte, *P* = 0.0034, 25.7 ± 11.9% vs. 37.2 ± 3.4%, *Lmna*^−/−^ and wt, in binuclear myocyte, *P* = 0.030, *n* = 7 in each group). EdU was injected intraperitoneally for 5 consecutive days from the day of birth and assessed at 21 days after birth. In the mononuclear myocyte population, EdU incorporated myocytes were also reduced in *Lmna*^−/−^ compared to wt (31.2 ± 16.3% vs. 62.7 ± 10.3%, *P* = 0.0047). EdU incorporation in both bi and polynuclear *Lmna*^−/−^ myocytes compared to wt was reduced but not significant. These results implicate that cardiomyocyte maturation is retarded because of the delayed cell cycle in *Lmna*^−/−^ mice.

### Reduced Cell Cycle Activity in Older *Lmna^+/−^* Mice

**Figure 3:**
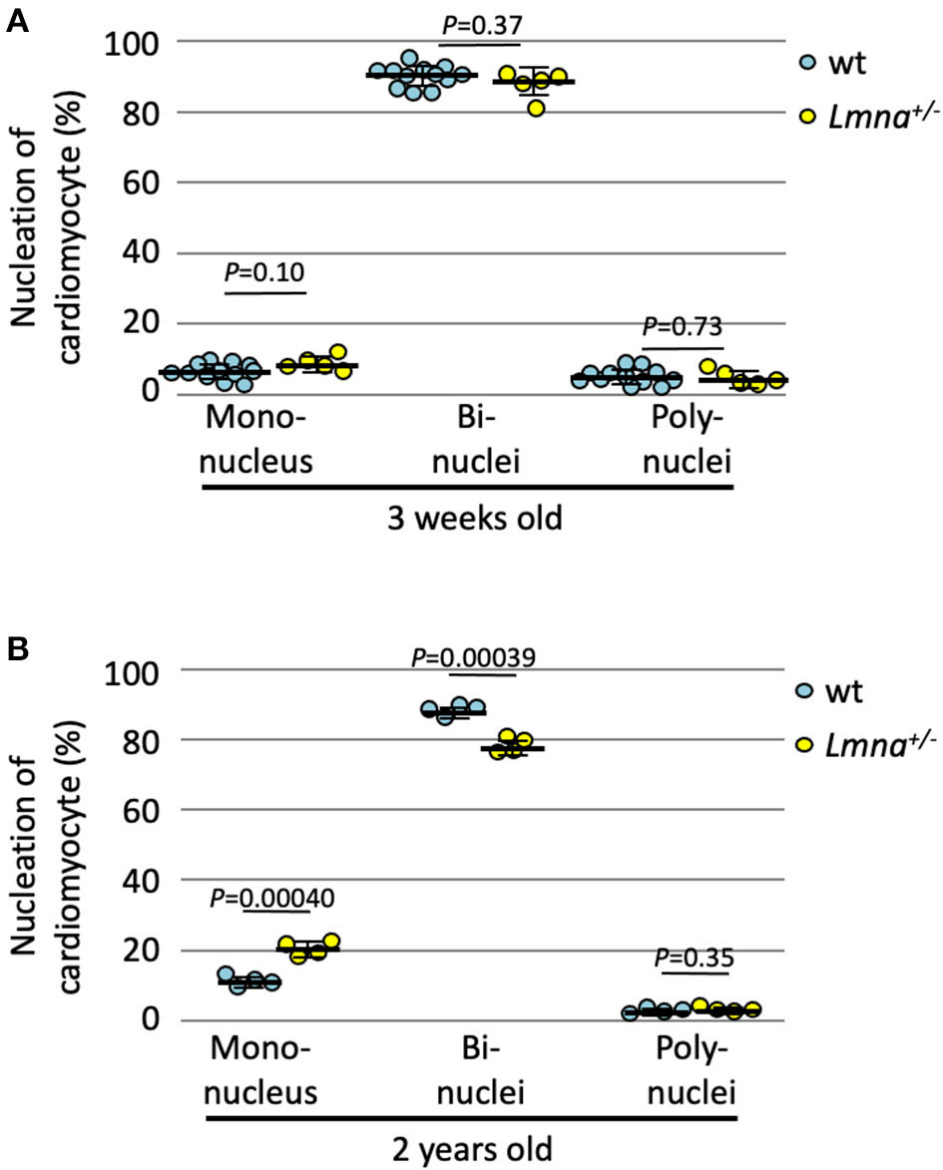
Nucleation of cardiomyocytes in young and old wild type (wt) and *Lmna*^+/−^ mice. **(A)** Nucleation of cardiomyocytes in 3 weeks old (*n* = 12 in wt and 5 in *Lmna*^+/−^) was similar between wt and *Lmna*^+/−^ mice. **(B)** Nucleation of cardiomyocytes in 2 years old (*n* = 4 in each group) showed more mononuclear and less binuclear cardiomyocytes in *Lmna*^+/−^ mice. Dots represent individual mouse data. The significance of differences between two groups was determined using the Student's 2 tailed *T*-test.

We also counted the number of nuclei per cell in 2-year-old *Lmna*^+/−^, which developed DCM phenotype, and wt mice. At 3 weeks of age, the nucleation was not different between wild type and *Lmna*^+/−^ mice (Figure 3A). In 2 years of age, however, *Lmna*^+/−^ mice had a significantly higher number of mononuclear myocytes compared to wt as same as observed in the younger generation of *Lmna*^−/−^ mice (19.8 ± 2.1% in *Lmna*^+/−^, 10.6 ± 1.5% in wt, *P* = 0.00040, *n* = 4 in each group, ~ 250 myocytes were counted in each mouse, Figure 3B), and binuclear cell counts were reduced in *Lmna*^+/−^ compared to wt (77.6 ± 2.2% in *Lmna*^+/−^, 87.3 ± 1.6% in wt, *P* = 0.00039, Figure 3B). The percentage of myocytes with more than 3 nuclei was not different between *Lmna*^+/−^ and wt in 2 years old (*P* = 0.35). These results suggest that insufficiency of *LMNA* reduces cell cycle activity and retards cardiomyocyte maturity throughout the lifetime.

### RNA-Sequencing of *Lmna* Mutant Mice

To elucidate the mechanism of reduced cell number, reduced cell size, immaturity, and cell cycle defects in *Lmna* mutant mice, we performed RNA-seq with RNA extracted from LV in *Lmna*^−/−^, *Lmna*^+/−^, and wt mice. One μg of RNA per mouse was pooled from 3 male mice and processed to construct a library as described in methods. Briefly, a total of 7 groups of mice were used: 5 weeks old wt, *Lmna*^+/−^, and *Lmna*^−/−^ mice, as well as wt and *Lmna*^+/−^ mice at 1 and 2 years of age. The numbers of reads processed after sequencing and alignment to the reference genome mm9 are shown in Supplementary Table 1. The number of reads aligned to each gene was normalized by the total number of aligned reads. We then compared the expression profile between *Lmna* mutant and age-matched wt mice in 4 groups; namely *Lmna*^−/−^/wt in 5 weeks, *Lmna*^+/−^/wt in 5 weeks, *Lmna*^+/−^/wt in 1 year, and *Lmna*^+/−^/wt in 2 years. Among 30,387 genes listed in mm9, more than 2,000 genes had altered expression profiles in *Lmna*^−/−^/wt in 5 weeks and *Lmna*^+/−^/wt in 2 years groups. In contrast, only <250 genes had altered expression profiles in *Lmna*^+/−^/wt in 5 weeks and *Lmna*^+/−^/wt in 1-year groups, which corresponds to the echocardiographic results before developing overt phenotype (the numbers of up- or down-regulated genes are shown in Supplementary Table 2). There were no up- or down-regulated genes overlapping across all 4 groups. We analyzed these data using Ingenuity Pathway Analysis (IPA), a pathway analysis software, to understand the biological functions of the genes with altered expression. The results are shown in Table 2 and Supplementary Table 3. We focused on the results of *Lmna*^−/−^ mice in 5 weeks old and of *Lmna*^+/−^ mice in 2 years old since those two groups develop similar cardiac phenotypes with different onset. We found a significant number of standard biological functions that have been impacted by this *Lmna* mutation. The “proliferation of cells” was identified as one of the categories that had a strong impact both on *Lmna*^−/−^/wt in 5 weeks and *Lmna*^+/−^/wt in 2 years groups. Supplementary Table 4 shows the genes in this category with a higher evidence level, associated with more than 50 papers reporting effects of these genes in cell proliferation. Among these genes, *Cdkn1a* had the most significant fold changes in both *Lmna*^−/−^/wt in 5 weeks and *Lmna*^+/−^/wt in 2 years groups, which encodes cell cycle regulating protein p21. Next, we analyzed which transcription regulators are mainly involved in *Lmna* mutation by IPA. As shown in Supplementary Table 5, TP53, which encodes p53 protein, was the most significantly affected transcriptional regulator in both *Lmna*^−/−^/wt in 5 weeks and *Lmna*^+/−^/wt in 2 years groups. As these genes exert significant effects in cell cycle regulation and proliferation, we inferred that upregulations of p53 and p21 contribute to the reduced cell proliferation and delayed cell cycle in *Lmna* mutant mice.

**Table 2:** Top biological functions related to *Lmna* mutant mice in RNA-seq analysis.

***Lmna**^**−/−**^*/**wt in 5 weeks**			***Lmna**^**+/−**^*/**wt in 2 years**		
**Biological function**	**Number of genes/total genes in the category**	***P*-value**	**Biological function**	**Number of genes/total genes in the category**	***P*-value**
Proliferation of cells	686/5,802	1.48E-33	Cell movement	429/3,105	8.54E-48
Cell death	652/5,975	3.7E-31	Migration of cells	394/2,784	2.24E-46
Apoptosis	550/4,663	4.16E-31	Proliferation of cells	620/5,802	1.54E-38
Necrosis	508/4,635	6.94E-27	Leukocyte migration	218/1,449	1.77E-32
Migration of cells	382/2,784	9.68E-26	Organization of cytoskeleton	269/1,685	3.35E-32
Cell movement	413/3,105	6.20E-25	Development of blood vessel	196/1,176	2.17E-30
Leukocyte migration	199/1,449	1.50E-17	Apoptosis	486/4,663	5.71E-30
Development of blood vessel	185/1,176	2.42E-17	Organization of cytoplasm	278/1,816	1.77E-29
Vascular disease	178/1,280	3.92E-17	Cell death	569/5,975	1.88E-28
Development of cardiovascular system	225/1,460	5.38E-17	Necrosis	460/4,635	2.48E-28

*Right-tailed Fisher's exact test*.

### p53 and p21 Are Highly Expressed; DNA Damage Is More Observed in *Lmna^−/−^* Mice

**Figure 4:**
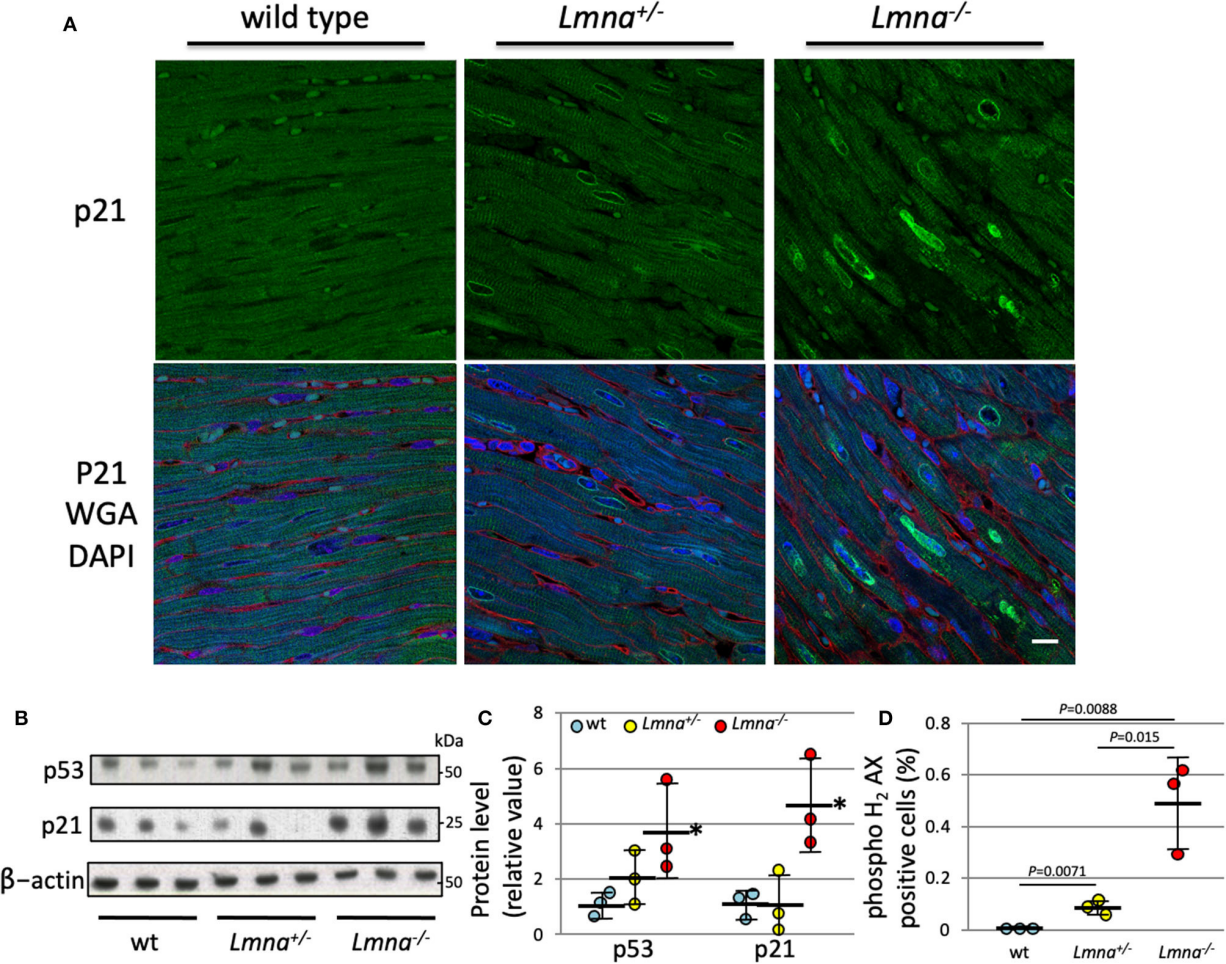
Protein expression of p21 and p53, and DNA damage of cardiomyocyte in 5 weeks. **(A)** Immunostaining against anti-p21 antibody in heart tissue section of wt, *Lmna*^+/−^, and *Lmna*^−/−^. The top panel shows p21 staining, and the bottom panel shows merged figures of anti-p21 antibody (green), WGA (red), and DAPI (blue). Scale bar: 10 μm. **(B)** Western blot analysis of LV tissue hybridized with p53, p21, and β-actin Abs. The data shown are representative of three independent experiments. **(C)** Protein expression level of p53 and p21 normalized by β-actin. *P < 0.05 vs. respective wt. **(D)** Percentage of phospho histone H_2_ AX staining counted in cardiomyocytes (Supplementary Figure 2) (*n* = 3 in each group). Dots represent individual mouse data. The significance of differences between 3 groups was determined with 1-way ANOVA. *Post-hoc* pairwise comparisons were performed with the Tukey–Kramer test.

We performed protein analysis to evaluate whether those proteins are also upregulated by *Lmna* mutation. As shown in Figure 4A, p21 was highly expressed in *Lmna*^−/−^ mice, especially in the nuclei of cardiomyocytes. Western blotting of protein extracted from LV revealed that not only p21 but p53 protein levels were also upregulated in *Lmna*^−/−^ mice with 4.7-fold for p21 (*P* = 0.012) and 3.7-fold for p53 (*P* = 0.030) compared to wt (Figures 4B,C).

As DNA damage is known to induce both p21 and p53 (35), we performed immunohistochemistry of phospho-histone H_2_AX (pH_2_AX) to detect DNA double-strand breaks (Supplementary Figure 2). We found a significantly higher percentage of pH_2_AX positive myocytes in *Lmna*^−/−^ compared to *Lmna*^+/−^ or wt mice (0.0043 ± 0.0011%, 0.069 ± 0.015%, and 0.49 ± 0.18%, wt, *Lmna*^+/−^ and *Lmna*^−/−^, respectively, Figure 4D). This result suggested that mice with *Lmna* mutation have more DNA damage than wt, possibly responsible for the increased p53 and p21 activities.

### *Lmna* Mutant Mice had a Repressed Cardiomyocyte Proliferative Capacity After Resecting the Apex of the Heart

**Figure 5:**
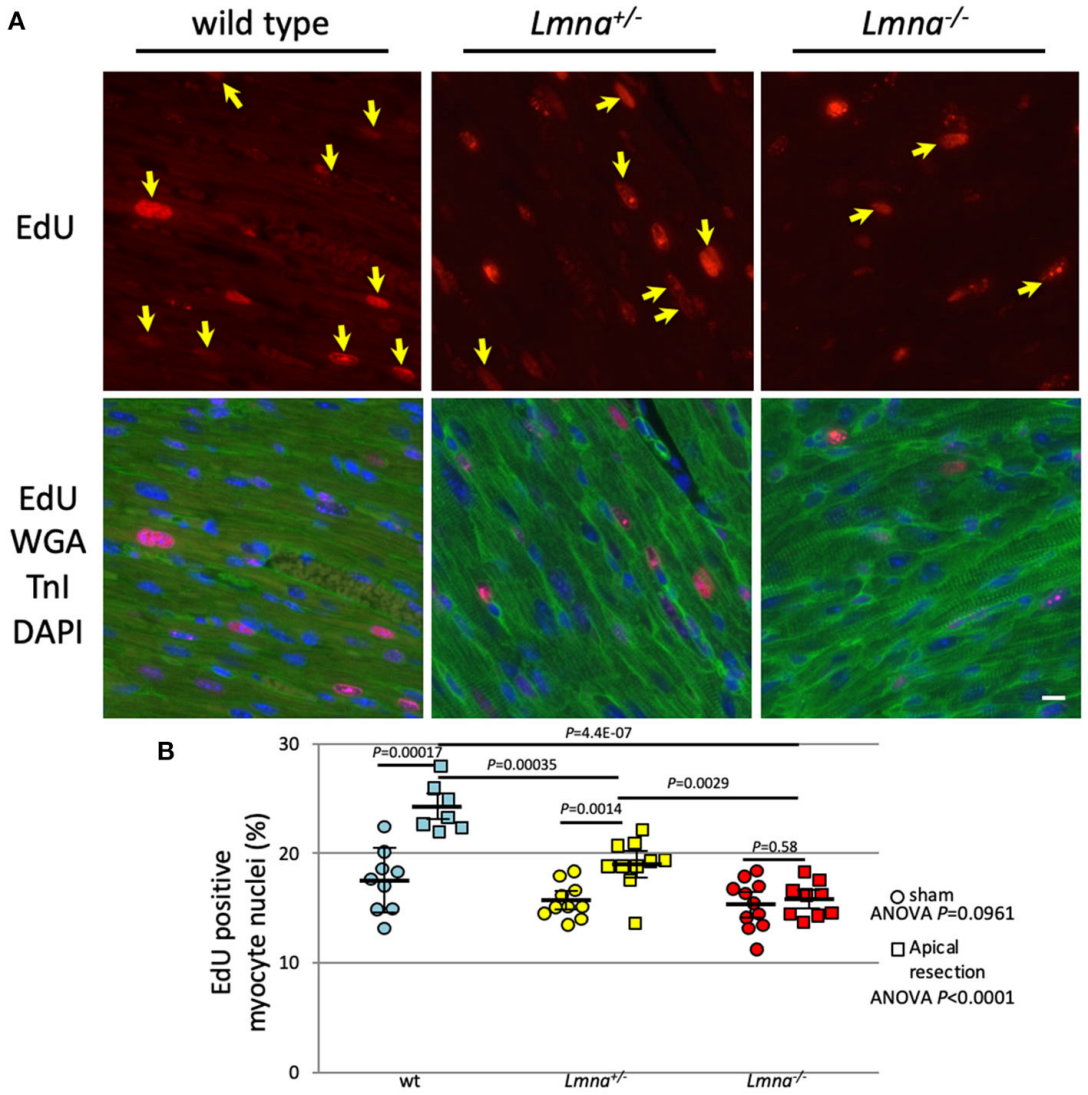
EdU incorporation and the percentage of EdU positive myocyte nuclei after apical resection. **(A)** Representative immunostaining for EdU in cardiac tissue sections of wt, *Lmna*^+/−^, and *Lmna*^−/−^. The top panel shows EdU staining, and the bottom panel shows merged figures of immunostaining for EdU (red), WGA, and troponin I (green) and DAPI (blue). Yellow arrows indicate EdU positive nucleus only in cardiomyocytes. Scale bar: 10 μm. **(B)** Percentage of EdU incorporated myocyte nuclei (*n* = 7 to 11 in each group). A circle indicates data from mice with the sham operation, square with apical resection. Dots represent individual mouse data. The significance of differences between 3 groups was determined with 1-way ANOVA. *Post-hoc* pairwise comparisons were performed with Tukey–Kramer test.

Next, we performed LV apical resection to assess the ability of cardiomyocyte proliferation. The experiment was performed on wt, *Lmna*^+/−^, and *Lmna*^−/−^ mice at the age of 2 days as described in Materials and Methods. EdU incorporated myocytes distributed around the resected area and remote area diffusely in the heart as previously reported (36). Eight high power fields per each cross-sectioned sample were chosen so that each field was evenly distributed across the section, and the percentage of EdU positive cardiomyocyte nuclei among total cardiomyocyte nuclei was counted. In the sham operation, the percentage of EdU positive cardiomyocyte nuclei was not different among 3 genotypes (17.5 ± 2.9%, 15.7 ± 1.6% and 15.3 ± 2.3%, wt, *Lmna*^+/−^, and *Lmna*^−/−^, respectively, ANOVA *P* = 0.0961, Figure 5). However, after apical resection, EdU incorporation into the nucleus was significantly accelerated, especially in wt from 17.5 ± 2.9% to 24.3 ± 2.2% (*P* = 0.00017). In *Lmna*^+/−^ mice, a similar effect was observed from 15.7 ± 1.6% to 19.1 ± 2.4% (*P* = 0.0014), although the change was milder than that in wt. On the other hand, in *Lmna*^−/−^ mice, the percentage of EdU positive cardiomyocyte nuclei did not increase from sham operation (15.3 ± 2.3%) even after apical resection (15.8 ± 1.6%, *P* = 0.58). The difference between these 3 groups after apical resection was significant (*P* < 0.0001). These data suggested that *Lmna* mutant mice have defective cardiomyocyte proliferative capacity compared to wt after stimulation, which normally leads to cell cycle activation.

## Discussion

We report here that the *Lmna* mutation retards cardiomyocyte proliferation and maturation processes and prevents compensatory proliferative response induced by apical resection, which could be one of the mechanisms of DCM development in patients with *LMNA* mutation.

It was reported that *Lmna* mutations are associated with DNA damage (12, 14, 37). As a structural protein of the nuclear envelope, the abnormality of lamin leads to deformity of the nuclear membrane and results in defective disassembly and reassembly processes of the nuclear envelope during the mitosis phase of the cell cycle. Since lamin also works as an anchor protein of chromatin, the abnormality of lamin further leads to defective chromatin arrangement (38, 39). These problems can influence DNA replication and thus be the cause for DNA damage (12, 14, 37). Once DNA is damaged, p53 and p21 are activated as previously reported (35, 40), which consequently retards the cell cycle and restricts cardiomyocyte proliferation as well as cardiomyocyte growth. In this study, DNA double-strand break was detected with pH_2_AX immunostaining, which can then activate the p53-p21 signaling axis. Actually, RNA-seq elucidated that p21 was most strongly activated in *Lmna* mutant mice, which was also confirmed by the protein expression. These results indicate that DNA damage, which arises from nuclear membrane malformation, structural and functional abnormalities as reported before, disturbs cell maturation and proliferation through the p53-p21 axis. Moiseeva et al. reported that depletion of *Lmna* leads to cell cycle arrest via p21 up-regulation by using primary human fibroblasts (29). In this study, we observed this cell cycle defects also *in vivo* heart as decreased cell numbers counted by tissue section.

Not only decreased cell numbers but also diminished cardiomyocyte binucleation was observed in *Lmna* mutant mice, suggesting inadequate maturation caused by cell cycle defects. As previously reported, mammalian cardiomyocytes exit the cell cycle after karyokinesis without cytokinesis shortly after birth (21, 24), making cardiomyocytes binucleated or multinucleated. The percentage of cardiomyocytes with more than 1 nucleus in adult varies depending on species, e.g., approximately 90% in mouse (21, 24), 70% in rat (41) and sheep (42), and 30% in human (43). In a murine heart, the higher the percentage of mononucleated cardiomyocyte exists, the more immaturity the cardiomyocyte has, because the ratio of a mononucleated cardiomyocyte is reported to be reduced as the heart develops (26). This decreased percentage of mononucleated cardiomyocyte was also observed in this study when we compared 8 days and 3 weeks old mice. The percentage of mononucleated cardiomyocytes was higher in *Lmna*^−/−^ than in wt mice in both ages. This fact also indicates cell cycle is retarded before the mitosis phase in *Lmna* mutant mice.

**Figure 6:**
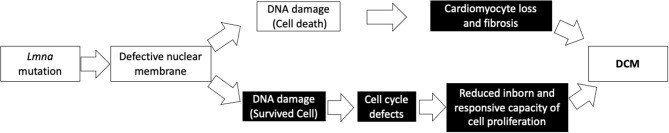
Our model depicting a possible mechanism in the development of DCM with a *Lmna* mutation. Black boxes denote findings from this study.

We propose that the vicious cascade of cell cycle defects in patients with *LMNA* mutations not only diminishes cell growth and proliferation in the physiological setting but alters the ability of the cell to respond against many kinds of stresses, including cardiomyocyte injury or damage, leading to the development of DCM. As shown in Figure 5, cardiomyocyte proliferative capacity after apical resection was diminished in *Lmna*^−/−^ mice. As Porrello et al. reported previously, once some signal concerning acceleration of cell proliferation is turned on, cardiomyocytes of mice can replicate in response to that if mice are not older than a week of age (31). Even after entering adulthood, cardiomyocytes can proliferate under stress, such as in ischemic heart disease (19). This compensatory myocyte proliferation was actually activated by apical resection in this study. However, the extent of DNA synthesis differed between wt and *Lmna* mutant mice. Furthermore, cardiomyocyte is reported to continuously but very slowly regenerate in human throughout life, 1% annually in young adult and 0.45% at the age of 75. And ~50% of cardiomyocytes are replaced during an average life span in humans (44). We, therefore, speculate that both the physiological regeneration process of cardiomyocytes during lifetime and the appropriate regenerative response under stress are retarded in patients with *LMNA* mutation, as suggested from the results of inappropriate cardiomyocyte maturation observed in 2-year-old *Lmna* mice and defective proliferative capacity after apical resection in *Lmna* mutant mice at 2 days after birth. And those defects could lead to the development of DCM, as observed in the reduced heart function at the age of 2 years in *Lmna*^+/−^ mice. Figure 6 shows our model of disease development for patients with *LMNA* mutations.

As we reported recently, cardiomyocytes in *Mybpc3* mutant mice also have fewer nuclei numbers (28). We concluded this is because cardiomyocytes undergo an additional round of cell division within 10 days after birth since MYBPC has inhibitory functions during postnatal cardiomyocyte cytokinesis and cell cycle progression. In *Mybpc3* mutant mice, DNA synthesis in cardiomyocytes and mitosis markers were observed in higher levels than wt mice, which is entirely opposite in *Lmna* mutant mice in this study. In *Mybpc3* mutant mice, the cell cycle progresses an additional round resulting in a higher percentage of mononuclear cardiomyocytes and a higher number of cardiomyocytes, whereas in *Lmna* mutant mice, cell cycle delays compared to wt resulting in a higher percentage of mononuclear cardiomyocytes but a fewer number of cardiomyocytes. This difference comes from a difference in the cell cycle, accelerated in *Mybpc3* and delayed in *Lmna* mutant mice, and presumably contributes to the phenotypic divergence of developing hypertrophic cardiomyopathy in patients with *MYBPC3* mutation while developing DCM in those with *LMNA* mutation.

We performed RNA-seq in this study, which resulted in robust transcriptional profiles (33, 45). We have successfully identified some biological functions that correlate with *Lmna* mutation. Among these, “cell proliferation” had the most prominent relationship to the *Lmna* mutation by the IPA pathway analysis. Indeed, we observed the cell number difference in physiological conditions as well as the repressed response after apical resection in *Lmna* mutant mice. Besides that, the biological functions related to “cell death” also significantly influenced *Lmna* mutant mice. It was difficult to identify the signal of cell death as either apoptosis or necrosis in this study, however, interstitial fibrosis in the heart was more frequently and massively observed in *Lmna* mutant mice, which suggested that injured cardiomyocytes were more abundant in *Lmna* mutant mice and were replaced by fibrosis. This progression of fibrosis could also be involved in DCM development with *Lmna* mutation (Supplementary Figure 1).

In this study, like many other studies, we found the body size and heart size difference between wt and *Lmna*^−/−^ mice, although their size is comparable when they are born. Lamin B is another structural protein in the inner nuclear membrane coded by *LMNB1*. *Lmnb1*^−/−^ mice are known to be embryonic lethal (46), while *Lmna*^−/−^ mice are not. In the embryonic stage, lamin B may have vital roles, and a class switch system from lamin B to lamins A and C at the perinatal stage may occur. Actually, when we compared the expression profiles in LV between embryonic day 14.5 (E14.5) and postnatal day 0, the expression of *Lmna* increases to 174.8%, while *Lmnb1* decreases to 34.8% of E14.5 mice at birth, respectively. This class switch of lamins might contribute to the cardiac growth defects after birth in *Lmna* mutant mice. Elucidation of the mechanism of the class switch system may also further reveal strategies to treat patients with *LMNA* mutation by either the inhibition of this class switch or by promoting the activation of the *LMNB1* gene.

Besides having a role in reinforcing the structure of the nucleus, lamin proteins are reported to be significant players in signal transduction or chromatin regulation (47, 48). Muchir et al. reported that mitogen-activated protein kinase (MAPK) signaling is activated in *Lmna* H222P knock-in mouse (*Lmna*^*H*222*P*/*H*222*P*^) hearts from abnormal activation of extracellular signal-regulated kinase (ERK) and Jun amino-terminal kinase (JNK), and this activation leads to DCM phenotype through actin depolymerization (49, 50). They also succeeded in proving that the inhibition of these pathways can prevent the mice from developing DCM (51). Another group showed only up-regulation of ERK and not of JNK after pressure overload induced by transverse aortic constriction (34). Our study did not observe any significant differences in these genes at any time point, which may be due to the different mouse lines between those previous studies and this study.

As the previous papers reported, conduction disorder and ventricular arrhythmia are also the characteristics of DCM with *LMNA* mutation (5, 17, 18). In most cases, the conduction disorder preceded LV dysfunction and ventricular arrhythmia (52, 53). In this study, although we didn't perform the precise evaluation on the arrhythmic status by electrocardiogram or telemetric studies as the previous study examined these electrophysiological properties using the same mutant mice line (17), we speculate the massive fibrosis more observed in *Lmna* mutant mice as shown in Supplementary Figure 1 could be the focus of ventricular arrhythmia. The reason why the conduction disorder precedes LV dysfunction or ventricular arrhythmia should be elucidated in a future study.

After the development, our model mice generated by the deletion of exon 8 to 11 of *Lmna* had been thought to be a “null” mutation (12). Recently Jahn et al. reported this mouse line has a truncated lamin A protein (54). Many researchers have used this *Lmna* mutant mouse line, and all studies are reported that this truncated protein does not work as a dominant-negative manner but as a loss-of-function manner (13, 17, 30, 55). Hence, we described this mouse line as *Lmna* knockout instead of *Lmna*^Δ8−11^ in this study. Although careful interpretation is needed to use this mouse line, we believe this truncated protein works as a loss-of-function manner because the heterozygous mice live as long as wt under normal conditions, although they develop heart dysfunction in the last stage of life.

In conclusion, we found that the cell cycle alterations, including activation of the p53-p21 axis and inadequate responses against stresses in *Lmna* mutant mice. We speculate these phenomena could play essential roles in the development of DCM caused by *LMNA* mutations. Although further studies are needed to fully understand the mechanism, modulating the cell cycle activity could be an efficient treatment strategy for DCM patients with *LMNA* mutation.

## Data Availability Statement

The data presented in the study are deposited in the NCBI BioProject repository, accession number PRJNA732812. This data can be found online at: http://www.ncbi.nlm.nih.gov/bioproject/732812.

## Ethics Statement

The animal study was reviewed and approved by Harvard Medical School and Nara Medical University.

## Author Contributions

KO designed the study, conducted and analyzed the experiments, performed the statistical analysis, and wrote the manuscript. HW contributed to study design, edited the manuscript, and conducted the animal experiments with JJ. MP and SD analyzed RNA-seq data. DC designed and conducted the animal study. JG contributed to extract RNA and build RNA-seq libraries. DM analyzed RNA-seq data, performed the statistical analysis, and edited the manuscript. JS, CS, and YS contributed to study design, provided scientific input, and edited the manuscript. All authors contributed to the article and approved the submitted version.

## Conflict of Interest

The authors declare that the research was conducted in the absence of any commercial or financial relationships that could be construed as a potential conflict of interest.
