# Supplementary material for: Cardiomyocyte Proliferative Capacity Is Restricted in Mice With Lmna Mutation
Source: Front Cardiovasc Med. 2021 Jun 23;8:639148. doi: 10.3389/fcvm.2021.639148 (PMC8260675; doi:10.3389/fcvm.2021.639148)
Supplement: Supplementary file 1 [file Data_Sheet_1.pdf]

Supplementary Material

Supplementary Data

Supplementary Tables

Supplementary Table 1. Number of reads processed and aligned to mouse genome (mm9)

	WT in 5 weeks	*Lmna*$^{+/-}$ in 5 weeks	*Lmna*$^{-/-}$ in 5 weeks	WT in 1 year	*Lmna*$^{+/-}$ in 1 year	WT in 2 years	*Lmna*$^{+/-}$ in 2 years
Total number of reads processed	155,841,948	147,171,682	140,488,230	71,536,068	77,191,076	69,896,038	74,310,846
Number of reads aligned to mouse genome (percentage of reads aligned)	122,041,795 (78.3%)	115,552,360 (78.5%)	110,397,124 (78.5%)	57,721,725 (80.7%)	61,518,741 (79.7%)	55,803,017 (79.8%)	57,377,011 (77.2%)

Supplementary Table 2. Number of up- or down-regulated genes in *Lmna* mutant mice among the total of 30,387 genes

	Number of up-regulated genes (ratio>1.5, *P*<0.001, normalized number of reads in WT>1)	Number of down-regulated genes (ratio<0.67, *P*<0.001, normalized number of reads in WT>1)
Lmna$^{-/-}$/WT in 5 weeks	1,654	1,092
Lmna$^{+/-}$/WT in 5 weeks	72	172
Lmna$^{+/-}$/WT in 1 year	135	30
Lmna$^{+/-}$/WT in 2 years	2,054	125

Supplementary Table 3. Top biological functions related to *Lmna* mutant mice from RNA-seq analysis.

***Lmna*$^{+/-}$/wt in 5 weeks**			***Lmna*$^{+/-}$/wt in 1 year**		
Biological function	Number of genes/total genes in the category	*P*-value	Biological function	Number of genes/total genes in the category	*P*-value
Extension of plasma membrane projections	8/197	4.55E-05	Activation of cells	34/1638	8.23E-14
Islet-cell carcinoma	3/15	1.21E-04	Quantity of blood cells	31/1506	3.01E-11
Abnormal morphology of parasympathetic postganglionic fiber	2/3	1.76E-04	Quantity of leukocytes	29/1322	3.02E-11
Loss of ciliary neuron	2/3	1.76E-04	Activation of blood cells	26/1183	7.29E-11
Diffuse B-cell lymphoma	5/110	1.78E-04	Activation of leukocytes	24/1014	1.47E-10
Development of digestive system	11/414	2.18E-04	Quantity of helper T lymphocytes	13/210	3.15E-10
Differentiation of muscle cells	9/314	2.90E-04	Leukocyte migration	26/1449	1.98E-09
Formation of cellular protrusions	17/1032	4.42E-04	Quantity of mononuclear leukocytes	23/995	4.34E-09
Survival of enteric neurons	2/6	5.79E-04	Proliferation of T lymphocytes	21/895	4.96E-09
Organization of cytoplasm	25/1886	6.41E-04	Encephalomyelitis	13/295	6.40E-09

Right-tailed Fisher's exact test.

Supplementary Table 4. Gene list involved in proliferation of cell category in RNA-seq analysis of *Lmna* mutant mice.

Gene	Description	Normalized reads in WT 5 weeks	Fold change *Lmna*$^{-/-}$/WT 5 weeks	Normalized reads in WT 2 years	Fold change *Lmna*$^{+/-}$/WT 2 years	Predicted effect to proliferation of cells in 5 weeks / 2 years	Number of evidence (papers)
Cdkn1a	cyclin-dependent kinase inhibitor 1	44.4	6.22	58.6	2.03	Decreased/Decreased	204
Agt	angiotensinogen	5.1	5.01	7.7	1.16	Increased/-	92
Igf1r	insulin-like growth factor I receptor	31.1	2.97	35.8	1.43	Increased/-	88
Myc	myc proto-oncogene protein	4.6	1.94	9.0	1.55	Increased/Increased	261
Egf	epidermal growth factor	8.2	-1.51	5.9	-1.29	Decreased/-	342
Cav1	caveolin-1	233.2	-1.59	192.3	1.02	Increased/-	58
Il15	Interleukin-15	2.7	-1.85	1.9	1.54	Decreased/Increased	207
Csf1	macrophage colony-stimulating factor 1	53.6	-2.02	47.5	1.02	Decreased/-	85
Tnfsf10	tumor necrosis factor ligand superfamily	6.3	-2.06	5.8	-1.05	Decreased/-	61
Kit	mast/stem cell growth factor receptor	3.5	-2.39	1.1	2.02	Decreased/Increased	106
Ccnd1	G1/S-specific cyclin-D1	34.8	-3.50	46.1	1.48	Decreased/-	86

Supplementary Table 5. Top transcription regulators related to *Lmna* mutation.

***Lmna*$^{-/-}$/WT in 5 weeks**			***Lmna*$^{+/-}$/WT in 2 years**		
Transcription regulators	Number of target genes in the category	*P*-value	Transcription regulators	Number of target genes in the category	*P*-value
TP53	229	6.63E-19	TP53	226	9.79E-28
MYC	161	1.94E-15	MYC	163	1.55E-23
STAT3	95	3.39E-15	MTPN	40	1.16E-20
NFKBIA	97	1.24E-13	NFKBIA	100	5.56E-20
CEBPB	86	1.28E-12	HTT	136	3.35E-18

Lmna$^{+/-}$/WT in 5 weeks			*Lmna*$^{+/-}$/WT in 1 year		
Transcription regulators	Number of target genes in the category	*P*-value	Transcription regulators	Number of target genes in the category	*P*-value
GATA2	5	3.12E-04	STAT3	15	3.97E-08
SP1	12	4.13E-04	CIITA	5	2.82E-06
NFATC4	3	1.09E-03	NFkB	15	4.01E-06
NFATC3	4	1.15E-03	STAT1	9	3.67E-05
CLOCK	5	6.51E-03	CTNNB1	13	5.18E-05

Right-tailed Fisher's exact test

Supplementary Figures

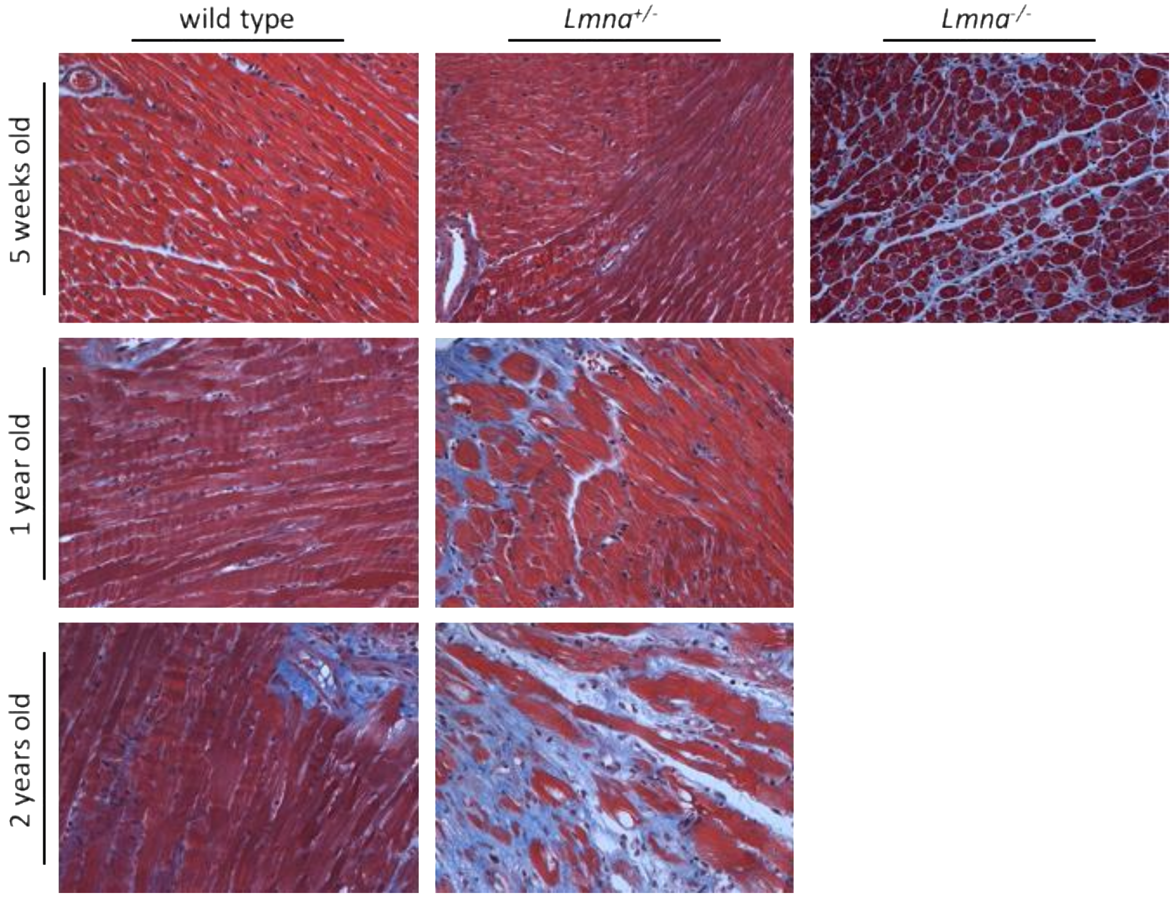

Supplementary Figure 1. Myocardial histology in mice aged 5weeks, 1 year, and 2 years stained by Masson-Trichrome staining.

Masson's trichrome staining showed massive fibrosis in *Lmna*$^{-/-}$ mice at 5 weeks of age. Interestingly, those cardiac phenotypes including enlarged LV chamber reduced contractile function, and severe fibrosis were comparable to those of *Lmna*$^{+/-}$ mice observed at 2 years of age (see also Table 1), which suggested that the cardiac phenotypes developed more rapidly in *Lmna*$^{-/-}$ mice than in *Lmna*$^{+/-}$ mice, sharing similar pathology at different ages. Scale bar: 50 µm.

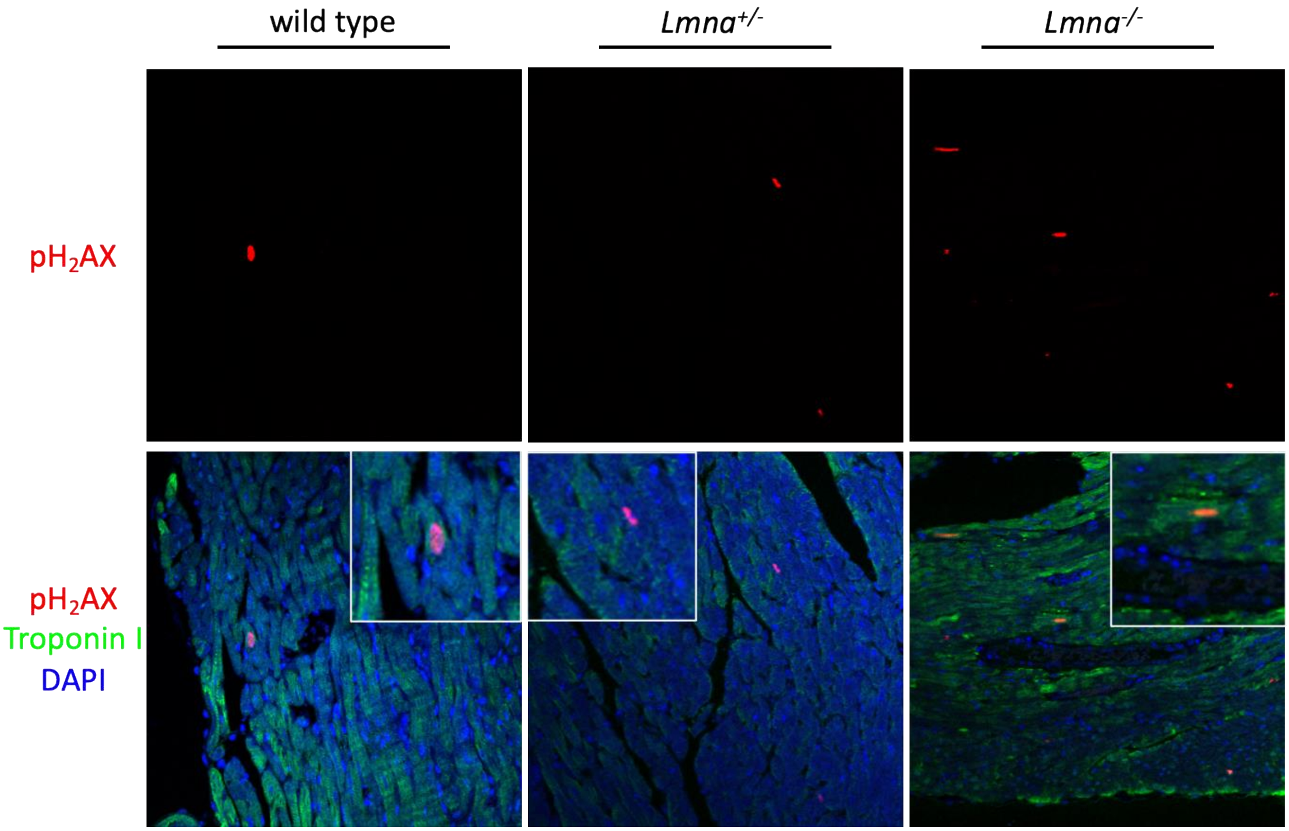

Supplementary Figure 2. Phospho Histone H_2AX staining in mice aged 5 weeks of wild type and *Lmna* mutations.

The top panel shows single immunostaining results, and the bottom panel shows merged figures of anti-pH_2AX Ab (red), anti-troponin I (green), and anti-DAPI (blue). Scale bar: 50 µm.

A significantly higher percentage of pH_2AX positive myocytes in *Lmna*$^{-/-}$ compared to *Lmna*$^{+/-}$ or WT mice were observed (see also Figure 4d). This result suggested that mice with *Lmna* mutation have more DNA damage than WT, possibly responsible for the increased p53 and p21 activities.

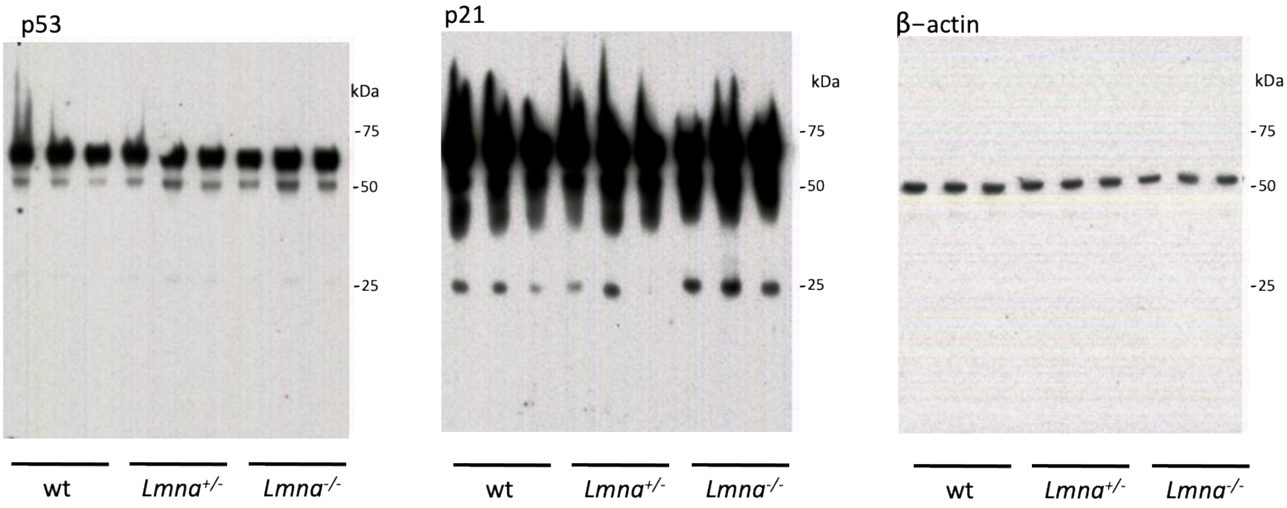

Supplementary Figure 3. Uncropped Blots of Figure4b. Protein expression of p21 and p53 in 5 weeks. Western blot analysis of LV tissue hybridized with p53, p21, and β-actin antibodies.
